# Supplementary material for: Virome-wide serological profiling reveals association of herpesviruses with obesity
Source: Sci Rep. 2021 Jan 28;11:2562. doi: 10.1038/s41598-021-82213-4 (PMC7843976; doi:10.1038/s41598-021-82213-4)
Supplement: Supplementary file 1 — Supplementary Information. [file 41598_2021_82213_MOESM1_ESM.pdf]

Virome-wide serological profiling reveals association of herpesviruses with obesity

Mohammad Rubayet Hasan[2,4#*], Mahbuba Rahman[1#], Taushif Khan[1#], Amira Saeed[2], Sathyavathi Sundararaju[2], Annaliza Flores[1], Philip Hawken[2], Arun Rawat[1], Naser Elkum[1], Khalid Hussain[1,4], Rusung Tan[2,4], Patrick Tang[2,4], Nico Marr[1,5]

[1]Department of Research, [2]Department of Pathology, [3]Division of Endocrinology,Department of Pediatrics, Sidra Medicine, Doha, Qatar; [4]Weill-Cornell Medical College, Doha, Qatar; [5]College of Health and Life Sciences, Hamad Bin Khalifa University, Doha, Qatar

***Corresponding author**

Mohammad Rubayet Hasan, PhD, FCCM, D(ABMM)

Assistant Professor of Clinical Pathology and Laboratory Medicine

Weill Cornell Medical College in Qatar (WCMC-Q)

Clinical Molecular Microbiologist, Department of Pathology, Sidra Medicine

Office no: H2M-24093, PO Box 26999, Doha, Qatar

Direct: +974 4003 2996; Mobile: +974 3003 5501

Email: mhasan@sidra.org

ORCID ID: 0000-0002-4658-7949

Supplementary Table 1: HSV-1 and -2 peptides significantly associated with obesity in the adult population

Cohort	Species	UniProt entry	Protein name	Peptide	Prev_Obese	Prev_Lean	log_or	log_pval
Adult	HSV1	P04288	Envelope glycoprotein M (gM)	PIYDEVADDQTDVLYAKIQHPRHLPDDDPIYDTVGGYDPEPAEDPVYSTVRRW	78.0	51.1	1.2	8.6
		P06484	Envelope glycoprotein G (gG) (gG-1)	VSSTTQPQLQTTGRPSHEAPNMTQTGTTDSPTAISLTTPDHTPPMPSIGLEEEEEE	80.2	56.5	1.1	7.1
		P06484	Envelope glycoprotein G (gG) (gG-1)	DSPTAISLTTPDHTPPMPSIGLEEEEEEEGAGDGEHLEGGDGTRDTLPQSPGPAFP	85.0	59.8	1.3	8.7
		Q69091	Envelope glycoprotein D (gD)	RRHTQKAPKRIRLPHIREDDQPSSHQPLFY	86.1	62.0	1.3	8.2
		Q8JQG9	Glycoprotein G (Fragment)	TPPMPSIGLEEEEEEEEGAGDGEHLEGGDGTRDTLPQSPGPAFPLAEDVEKDKPNRP	85.7	60.9	1.3	8.7
		Q8JQR0	Glycoprotein G (Fragment)	DGTRDTLPQSPGPAVPLAGDDEKDKPNRPVVPPPGPNNSPARPETSRPKHPPPVSG	79.8	54.3	1.2	8.1
		Q8JQS3	Glycoprotein G (Fragment)	TPPMPSIGLEEEEEEEEEEGAGDCEHLKGGDGTRDTLPQSPGPAVPLAGDDEKDKPN	83.9	58.7	1.3	8.5
		Q8JQS3	Glycoprotein G (Fragment)	GDGTRDTLPQSPGPAVPLAGDDEKDKPNRPVVPPPGPNNSPARPETSRPKTPPTSI	72.9	50.0	1.0	6.0
		P06437	Envelope glycoprotein B (gB) (gB-1) (gB1)	PPLGAAPTGDPKPKKNKKPKNPTPPRPAGDNATVAAGHATLREHLRDIKAENTDAN	72.9	47.8	1.1	7.1
		P08665	Envelope glycoprotein B (gB) (gB-1) (gB1)	SAAPSSPGTPGVAAATQAANGGPATPAPPALGAAPTGDPKPKKNKKPKNPTPPRPA	71.1	44.6	1.1	7.7
		P08665	Envelope glycoprotein B (gB) (gB-1) (gB1)	PALGAAPTGDPKPKKNKKPKNPTPPRPAGDNATVAAGHATLREHLRDIKAENTDAN	74.4	49.5	1.1	7.2
		P36318	Envelope glycoprotein D (gD)	RRRTQKGPKRIRLPHIREDDQPSSHQPLFY	82.0	60.9	1.1	6.0
		P06476	Envelope glycoprotein D (gD)	RRTRKAPKRIRLPHIREDDQPSSHQPLFY	81.3	59.2	1.1	6.4
	HSV2	P89433	Envelope glycoprotein M (gM)	APDHEAELYARVQRPGPVPDAEPIYDTVEGYAPRSAGEPVYSTVRRW	39.2	18.5	1.0	5.7

Supplementary Table 2: HSV-1 and -2 peptides associated with obesity in both adult and pediatric population

Cohort	Species	UniuProt entry	Protein name	Peptide	Prev_Obese	Prev_Lean	log_or	log_pval
Adult	HSV1	F8RDH2	Tegument protein US11	MSQTQPPAPVGPGDPDVYLKGVPSAGMH**PRGVHAPRGHPRMISGPPQRG**DNDQAAG	45.8	27.7	0.8	3.9
Pediatric	HSV1	F8RDH2	Tegument protein US11	**PRGVHAPRGHPRMISGPPQRGDNDQAAGQ**CGDSGLLRVGADTTISKPSEAVRPPTF	23.4	10.8	0.9	1.8
Pediatric	HSV1	P04487	RNA-binding protein (Vmw21)	**PRGVHAPRGHPRMISGPPQRGDNDQAAG**QCGDSGLLRVGADTTISKPSEAVRPPTI	20.9	9.9	0.9	1.5
Pediatric	HSV2	P89466	Tegument protein UL46 (Tegument protein VP11/12)	AAWPAESHAPRAPSEDADSIYESVGEDGGRVYEEIPWVRVYENICPRRRLAGGAAL	18.4	7.2	1.1	1.8
Adult	HSV2	P89466	Tegument protein UL46 (Tegument protein VP11/12)	AAWPAESHAPRAPSEDADSIYESVGEDGGRVYEEIPWVRVYENICPRRRLAGGAAL	18.7	7.1	1.1	3.5

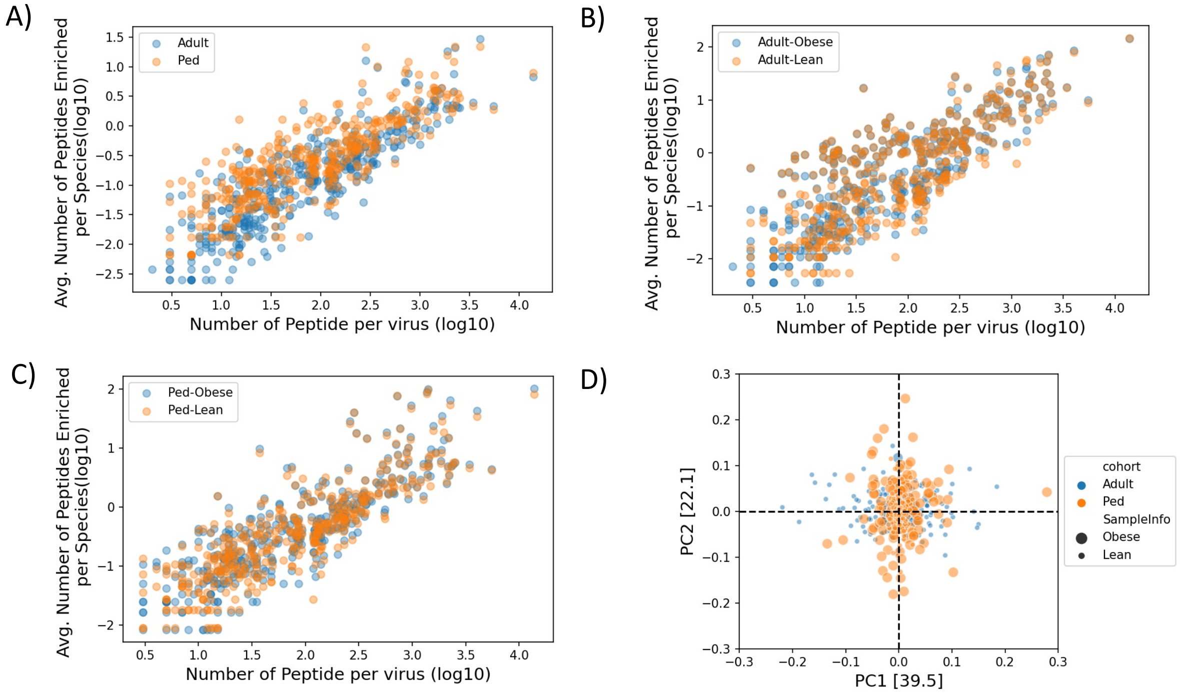

Supplementary Figure 1: Enrichmnet profile of peptides with respect to virus library size (Species wise) in adult and pediatric obese and lean groups. Number of peptides in the VirScan library for different viral species (log transformed) in the x-axis plotted against species wise average number of enriched peptides: (A) Adult vs Pediatric cohort (B) Obese versus Lean in adult cohort and (C) Obese versus Lean in the pediatric cohort. (D) Principal component analysis of enriched peptides in obese and lean samples from adult and pediatric cohorts. Here, scatter plot of first two principal components (PC1 and 2) has been shown that describes 57% of variance in peptide enrichment profiles.

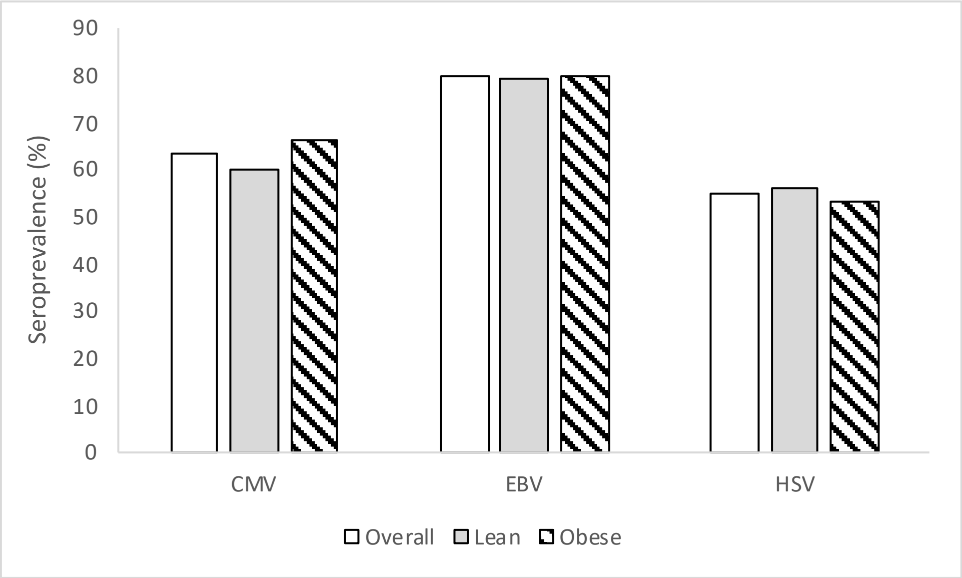

Supplementary Figure 2: Seroprevalence of herpes viruses in the pediatric obese and lean population by standard serological methods (N, obese = 120; N, lean = 111)

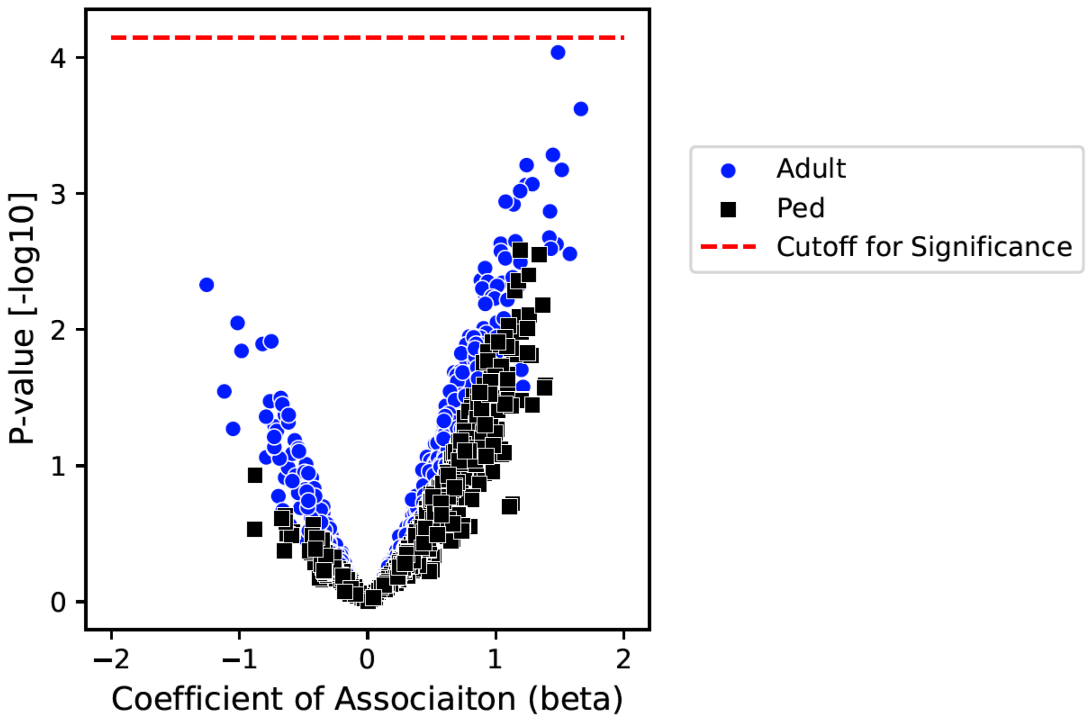

Supplementary Figure 3: Association of any combination of two viruses with obesity. A total of 43 viral species that are at least 10% prevalent in the overall study population were tested for their association with obesity in the adult and pediatric population by multiple logistic regression analysis, with age and gender as co-variates, and using a two-way interaction model. The co-efficient of association (x-axis) of any combination of two viruses were plotted against respective *p*-values (-log10) (y-axis) after Bonferroni correction for multiple testing. The red dotted line indicates significance threshold after Bonferroni correction. HSV1, herpes simplex virus 1; RVA, rotavirus.
